# Acupuncture clinical hotspots and trends from 2013 to 2022: A bibliometric and visualized analysis

**DOI:** 10.1097/MD.0000000000041890

**Published:** 2025-04-04

**Authors:** Xiangdong Wang, Bo Li, Yuping Ma, Ying Cui, Xinming Yang, Yuxian Li, Anna Jing, Yutong Zhou, Mingyue Li, Sixuan Wang, Yufeng Tu

**Affiliations:** a First Teaching Hospital of Tianjin University of Traditional Chinese Medicine, Tianjin, PR China; b National Clinical Research Center for Chinese Medicine Acupuncture and Moxibustion, Tianjin, PR China; c Tianjin University of Traditional Chinese Medicine, Tianjin, PR China; d Shandong Provincial Hospital (Group) Jinan Beicheng Hospital, PR China.

**Keywords:** Acupuncture, bibliometrics, CiteSpace, methodological, VOSviewer

## Abstract

Through bibliometric analysis, trends in international acupuncture clinical development, key research areas, and current scientific issues were identified based on literature published from 2013 to 2022. Literature on acupuncture in clinical settings was retrieved and analyzed in this study utilizing the Web of Science database. A visualization analysis of the scientific landscape was performed using CiteSpace, VOSviewer, and GraphPad Prism. General statistics regarding the literature were examined, encompassing annual publication trends, citation frequencies, journal distributions, distributions across subject fields. Co-occurrence and cluster analyses of authors, countries, institutions, high-quality literature, and keywords were performed to explore the developmental trends, hotspots, and frontiers in acupuncture research comprehensively and intuitively. A total of 4554 studies were included, with an increasing trend in the number of publications related to the annual acupuncture Scientific Citation Index (SCI). The impact factors of the top 10 journals were mostly 1 to 3 points. The 10 most cited articles primarily concentrated on the use of acupuncture for pain-related disorders. Acupuncture and electroacupuncture are the dominant keywords, with terms such as “ischemic stroke” and “Alzheimer’s disease” emerging from 2019 to 2022. From 2013 to 2022, the output of clinical SCI literature on acupuncture increased, acupuncture research gradually gained international recognition, and China gradually moved toward a dominant position. The most significant clinical research area of acupuncture is its application for analgesia, particularly through acupuncture and electroacupuncture. Future research directions may include the treatment of knee osteoarthritis, cognitive disorders, and assisted reproduction utilizing acupuncture.

## 1. Introduction

Acupuncture, a traditional therapeutic practice in China, is widely utilized across diverse medical fields. The Yellow Emperor’s Inner Canon contains specific chapters on acupuncture treatments for over 100 diseases. Ongoing global research conducted by acupuncture scholars has broadened its application to 16 systems and has addressed a total of 532 diseases. This growing breadth of research highlights the extensive potential of acupuncture in medical practice. Acupuncture is currently practiced in 196 countries and regions, and it has been incorporated into the mainstream healthcare systems of nations such as the United States, South Korea, Germany, and Australia, where its utilization is supported by national guidelines and insurance coverage. This widespread integration highlights the significance of acupuncture across various clinical settings and cultural contexts. Additionally, substantial progress has been made in its clinical application for managing chronic pain, cancer, and related complications, demonstrating its broad and promising global potential.^[1]^ The World Health Organization, through its Traditional Medicine Strategy, has emphasized the necessity of international collaboration to advance acupuncture research and has established a comprehensive policy framework to support its global development.^[2]^ This increasing international recognition underscores the importance of analyzing global research trends in acupuncture to enhance understanding of its evolution, efficacy, and impact across diverse healthcare systems and cultural backgrounds.

Building on this global perspective, recent clinical research on acupuncture has focused predominantly on key areas such as mechanistic explanations, efficacy predictions, and the evaluation of suitable patient populations.^[3,4]^ Additionally, progress in modern biomedical technologies, such as neuroimaging, systems biology, and molecular biology, has increasingly facilitated advancements in acupuncture research, resulting in significant achievements.^[5,6]^ Over the past thirty years, a continuous accumulation of literature on acupuncture has occurred, with substantial interest from both domestic and international research institutions, especially in the last decade, focusing on its scientific and clinical applications. As research fields expand, trends and focal points in acupuncture are gaining increased interest among scholars globally.^[7]^

Scientometrics and scientometric analysis efficiently extract relevant information from exponentially growing data sources, facilitating the retrieval of databases and topics related to scientific research. Through the analysis, quantification, and summarization of these data, objective development patterns in related fields and disciplines can be identified.^[8,9]^ Visualization software, including CiteSpace and VOSviewer, has innovatively utilized information visualization techniques in scientometrics, emerging as widely used tools in the field.^[10]^ In acupuncture research, scholars widely recognize and utilize visualization software for literature analysis.^[11–15]^ Nevertheless, extensive studies that have been published over the past decade that employ visualization software to analyze acupuncture-related clinical Scientific Citation Index (SCI) are lacking. This study applies bibliometric theory, spatiotemporal analysis, and content knowledge graph analysis as the research methodologies. Using CiteSpace and VOSviewer for literature visualization, we analyzed acupuncture-related clinical SCI literature published from 2013 to 2022. The aim is to summarize international trends in acupuncture clinical research, key focus areas, and prominent scientific issues, offering a foundation and direction for future high-quality research and innovative developments in acupuncture clinics worldwide. In recent years, bibliometric and visualized analyses of acupuncture research have gained increasing attention. For example, the study titled “A Comprehensive Overview of Acupuncture Therapy Over the Past 20 Years: Machine Learning-Based Bibliometric Analysis” utilized machine learning techniques to conduct an in-depth analysis of the themes and hotspots of scientific publications on acupuncture therapy over the past 20 years.^[16]^ This study employed latent Dirichlet allocation and the Louvain algorithm for topic network analysis, comprehensively revealing the research trends of acupuncture therapy as well as the research status and hotspots in different countries. Another study, “Bibliometric and Visualized Analysis of Electroacupuncture in the Past 10 Years,” focused on the subfield of electroacupuncture, identifying research hotspots and developmental trends.^[17]^ These studies have provided valuable insights into acupuncture research, especially in long-term trend analysis, in-depth exploration of specific areas, and the application of advanced methods such as machine learning. However, given the rapid development in the field of acupuncture research, particularly in clinical applications, there is a need for more detailed and up-to-date analyses to reflect the current research dynamics. Therefore, the uniqueness of this study lies in its focus on the past decade (2013–2022) and its exclusive focus on clinical research. Through multidimensional analysis and the use of advanced tools such as CiteSpace, VOSviewer, and GraphPad Prism, this study offers a novel perspective on the evolving academic landscape of acupuncture clinical research. This study provides a comprehensive overview of recent trends and advancements while emphasizing the global significance of acupuncture.

## 2. Materials and methods

### 2.1. Data sources and search strategies

A thorough search of the Science Citation Index Expanded Journal Database in the Web of Science (WOS) was performed from January 1, 2013, to December 31, 2022. All searches were completed on April 1, 2023, to eliminate bias from database updates. The retrieval strategy was as follows: TS =((Acupunctur*) OR (acupoint*) OR (Electroacupunctur*) OR (electr*-acupunctur*) OR (fire needl*)) AND Language: (English) AND Document type: (Article OR Review), IC Timespan = 2013–2022. The index is set to the Science Citation Index Expanded (SCI-EXPANDED).

### 2.2. Inclusion and exclusion criteria for the literature review

We included journal articles from the WOS database related to clinical research on acupuncture, which the SCI has indexed. The language was limited to English. We excluded animal experimental studies, laser acupuncture, acupoint injection, transcutaneous electrical stimulation, magnetic stimulation, acupressure, acupoint application, massage therapy, moxibustion, cupping therapy, books, guidelines, consensus statements, case discussions and reports, news articles, advertisements, calls for published literature, duplicated publications, and incomplete data.

### 2.3. Statistical and analytical methods

Quantitative analysis and research were conducted on external features such as the number of publications, distribution by publication year, citation frequency, journal distribution, funding sources, and keywords from 2013 to 2022. The data were imported into 3 literature visualization analysis software programs: CiteSpace 6.2. R6, VOSviewer 1.6.19, and GraphPad Prism 8.3. Co-occurrence visualization analyses were performed using these platforms on authors, countries, institutions, highly cited articles, and keywords.

## 3. Results

### 3.1. Annual inclusion of acupuncture clinical research papers in the SCI literature

**Figure 1.**
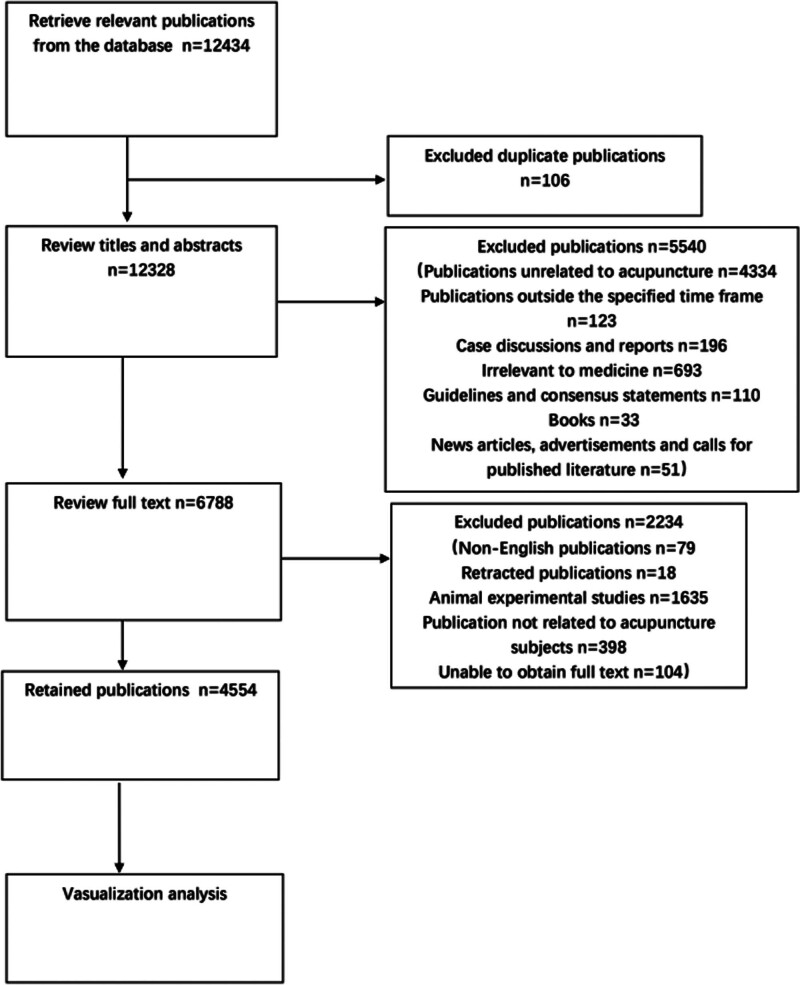
Flow chart of data selection.

**Figure 2.**
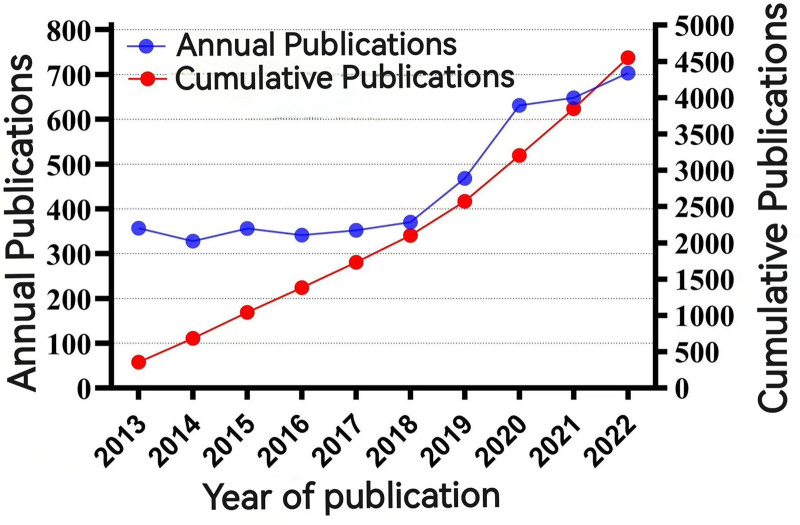
Trend chart of the annual inclusion of acupuncture clinical studies on SCI from 2013 to 2022. SCI = Scientific Citation Index.

Utilizing the defined search scope and retrieval strategy, a total of 12,434 articles were initially identified. Following the exclusion of 7880 articles, 4554 were considered relevant for analysis. The detailed process of article selection is presented in Figure 1. As illustrated in Figure 2 and Table 1, the number of publications pertaining to acupuncture-related clinical SCIs showed a consistent increase from 2013 to 2022. The lowest number of publications occurred in 2014 (328 articles, 7.20%), whereas the highest was in 2022 (703 articles, 15.44%). On average, 455.4 articles were published annually. A noticeable shift occurred in 2018, when the publication trend shifted from stable to significantly increasing. Although the growth rate slowed from 2020 to 2022 compared with earlier years, it still represented substantial progress from before 2018.

**Table 1:** Annual number of acupuncture clinical SCI studies included from 2013 to 2022

Year of publication	Number of literature	Percentage
2013	357	7.84
2014	328	7.20
2015	356	7.82
2016	341	7.49
2017	352	7.73
2018	370	8.12
2019	468	10.28
2020	631	13.86
2021	648	14.23
2022	703	15.44

SCI = Scientific Citation Index.

### 3.2. Distribution of globally indexed clinical acupuncture SCI journals

The journal impact factors are obtained from http://www.medsci.cn/sci/, which was accessed on June 5, 2023.

The literature included in the study spans 679 journals, with the top 10 journals contributing 2050 publications, accounting for 45.50% of all the articles. The journal “Evid Based Complement Alternat Med” led with 486 indexed articles, accounting for 10.67% of the total. The majority of journals are based in the United Kingdom and include 3 different journals. The impact factors for these journals generally range from 1 to 3, with “PLoS One” exhibiting the highest impact factor (IF 3.852) and “Eur J Integr Med” displaying the lowest (IF 1.813). The journal quartile distribution is mainly in Q2 and Q3, with only one journal falling into Q1. Detailed information can be found in Table 2.

**Table 2:** Top 10 journals publishing clinical acupuncture studies indexed in SCI from 2013 to 2022

Publication title	Number of included literature	Proportion of literature	Subordinate country	Impact factor	JCR partition
Evid Based Complement Alternat Med	486	10.67%	United Kingdom	2.65	Q3
Medicine	479	10.52%	United States	1.817	Q3
Acupunct Med	300	6.59%	United Kingdom	1.976	Q3
Trials	216	4.74%	United States	2.728	Q3
J Altern Complement Med	123	2.70%	United States	2.381	Q3
BMC Complement Med Ther	117	2.57%	United Kingdom	2.838	Q1
BMJ Open	111	2.44%	United States	3.006	Q2
Eur J Integr Med	84	1.84%	Netherlands	1.813	Q2
PLoS One	81	1.78%	United States	3.852	Q2
Chin J Integr Med	75	1.65%	China	2.626	Q2
Total	2050	45.50%			

SCI = Scientific Citation Index.

### 3.3. Distribution of SCI publications in the Chinese acupuncture clinical literature

The top 10 journals, ranked by the number of SCI clinical acupuncture articles published by Chinese scholars in the past decade, are presented in Table 3. The total number of articles published in these top 10 journals was 1409, representing 30.94% of the total number of included articles. The top 3 journals are Medicine (419 articles, 9.20%), Evid-Based Complement Alternat Med (348 articles, 7.64%), and Trials (162 articles, 3.56%). A comparison of the top 10 journals by global SCI articles (Table 2) revealed that 3 journals, J Tradit Chin Med, J Pain Res, and Front Neurosci, are among the top 10 Chinese SCI articles. In contrast, 3 journals, Eur J Integr Med, J Altern Complement Med, and PLoS One, are not included in the list. Among them, J Tradit Chin Med and Chin J Integr Med are journals from China, and the number of Chinese SCI articles published in them accounts for 2.66% of the total number of included articles.

**Table 3:** Top 10 Chinese journals publishing clinical acupuncture studies indexed in SCI from 2013 to 2022

Publication title	Number of included literature	Proportion of literature	Subordinate country	Impact factor	JCR partition
Medicine	419	9.20%	United States	1.817	Q3
Evid Based Complement Alternat Med	348	7.64%	United Kingdom	2.65	Q3
Trials	162	3.56%	United Kingdom	2.728	Q3
Acupunct Med	117	2.57%	United Kingdom	1.976	Q3
BMJ Open	85	1.87%	United Kingdom	3.006	Q2
J Tradit Chin Med	63	1.38%	China	2.547	Q3
BMC Complement Med Ther	58	1.27%	United Kingdom	2.838	Q1
Chin J Integr Med	58	1.27%	China	2.626	Q2
J Pain Res	50	1.10%	United Kingdom	2.832	Q3
Front Neurosci	49	1.08%	Kingdom of Sweden	5.152	Q2
Total	1409	30.94%			

SCI = Scientific Citation Index.

The journal impact factors are obtained from http://www.medsci.cn/sci/, which was accessed on June 5, 2023.

### 3.4. Global distribution of authors and collaborative networks in the clinical acupuncture SCI literature

A total of 16,709 authors were included in the included studies, which were ranked in descending order by citation frequency. The general information and research directions of the top 10 most cited authors are presented in Table 4. Liang and Fangrong have achieved the highest total citation frequency, amounting to 1639 for their publications in the past decade. Mao, Jun J, ranked 10th, has received 837 citations for his published work. Among the top 10 authors listed, Liu, Zhishun (with 101 papers), Liang, Fanrong (98 papers), and Liu, Cunzhi (72 papers) held the top 3 positions in terms of publication volume over the past decade, demonstrating expertise in their respective research fields. Notably, Klaus Linde from the Technical University of Munich, Germany, has received 1017 citations, despite having published only 11 papers in the last decade. These authors exhibit distinct and specialized research directions, each establishing a unique academic footprint in their respective fields.

**Table 4:** Top 10 authors with the highest citation frequencies of acupuncture clinical SCIs from 2013 to 2022

Author	Number of citations	Number of Published Articles	The country	Associated entities	Field of research
Liang, Fanrong	1639	98	China	Chengdu University of Traditional Chinese Medicine	Research on the clinical efficacy and biological mechanisms of acupuncture points, evidence-based methodology for acupuncture, neuroimaging in acupuncture, metabolomics, and sensitization of acupoints
Liu, Zhishun	1353	101	China	China Academy of Chinese Medical Sciences Guang’anmen Hospital	Investigation of lower urinary tract dysfunction, chronic pain acupuncture diagnostics and treatment standards, as well as the underlying mechanisms, alongside fundamental research into the clinical practice of acupuncture
Lao, Lixing	1333	45	United States	University of Maryland School of Medicine	The efficacy and mechanism of acupuncture in treating inflammatory pain.
Kong, Jian	1161	26	United States	Massachusetts General Hospital	Brain imaging techniques are used in both clinical and basic research to explore the central mechanisms of acupuncture.
Liu, Cunzhi	1161	72	China	Capital Medical University	Specific effects of acupoints and their antioxidant mechanisms.
Li, Ying	1136	55	China	Chengdu University of Traditional Chinese Medicine	Clinical Research on Advantage Diseases of Acupuncture and Moxibustion and Research on Its Mechanism of Action
Macpherson, Hugh	1090	30	United Kingdom	York University	Development of Acupuncture Practice Guidelines and Exploration of RCT Principles and Methods
Linde, Klaus	1017	11	Germany	Technical University of Munich, Germany	Randomized controlled trials (RCTs) for acupuncture treatment of various chronic pain conditions.
Lee, Myeong Soo	1012	50	South Korea	Korean Oriental Medical College	A retrospective study on the clinical efficacy of acupuncture, the development of evidence maps and reporting guidelines, and the formulation of clinical practice guidelines.
Mao, Jun J.	837	34	United States	The University of Pennsylvania	Clinical and Mechanistic Studies on Acupuncture for Cancer-Related Pain

SCI = Scientific Citation Index.

**Figure 3.**
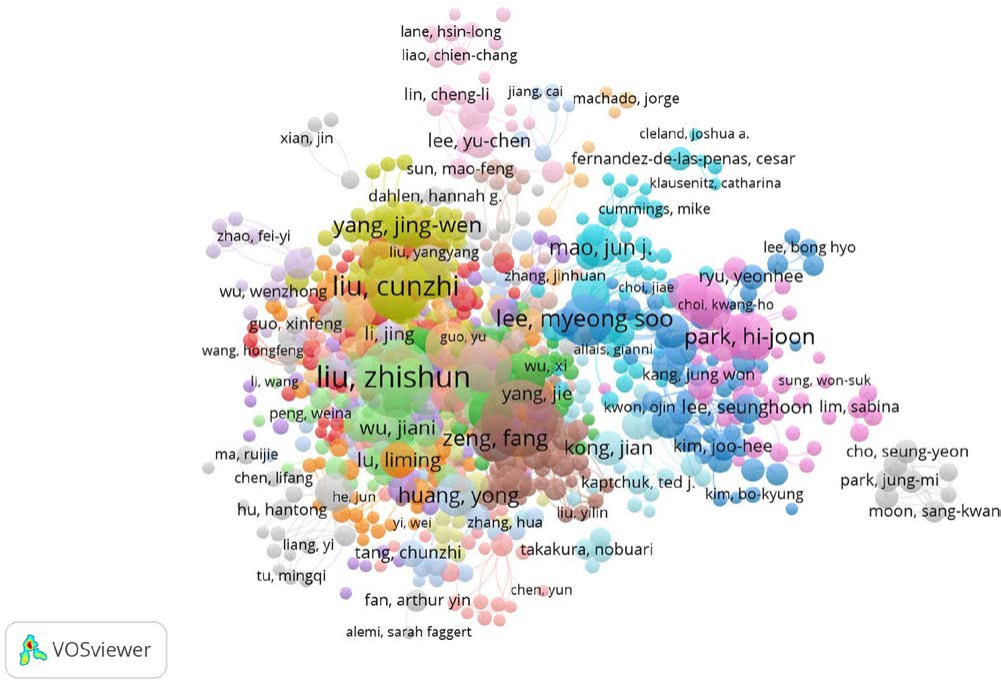
Cocitation network map of SCI authors involved in acupuncture clinical research from 2013 to 2022. SCI = Scientific Citation Index.

Setting VOSviewer version 1.6.19’s minimum author contribution parameter to 10 reveals a coauthorship network map featuring 232 contributing authors, as depicted in Figure 3. In this visualization, circular nodes represent individual authors, scaled by their publication volume. Line thickness represents the strength of collaborative links, emphasizing enhanced partnerships among these scholars. The colored clusters signify closely connected research teams. Notably, Liu Zhishun and Liang Fanrong exhibit comparable node sizes, each with 101 and 98 publications, respectively. Liang Fanrong stands out with a total link strength of 343, actively collaborating with 49 researchers, including Li Ying, Zeng Feng, and Zhao Ling. Liu Zhishun shows substantial collaboration with a link strength score of 319, involving 52 peers. Overall, researchers in acupuncture SCI have developed intricate and stable collaboration networks, as illustrated by teams led by Liu Cunzhi, Liu Zhishun, and Liang Fanrong in the Chinese author collaboration region located in the lower left of the figure. Simultaneously, the upper right represents international researcher collaboration, featuring teams led by Lee Myeong-soo, Park Hi-joon, Mao Jun-j, and Lee Yu-Chen, highlighting robust domestic and international cooperation in acupuncture research and its global impact.

### 3.5. Chinese distribution of authors and collaborative networks in the clinical acupuncture SCI literature

**Figure 4.**
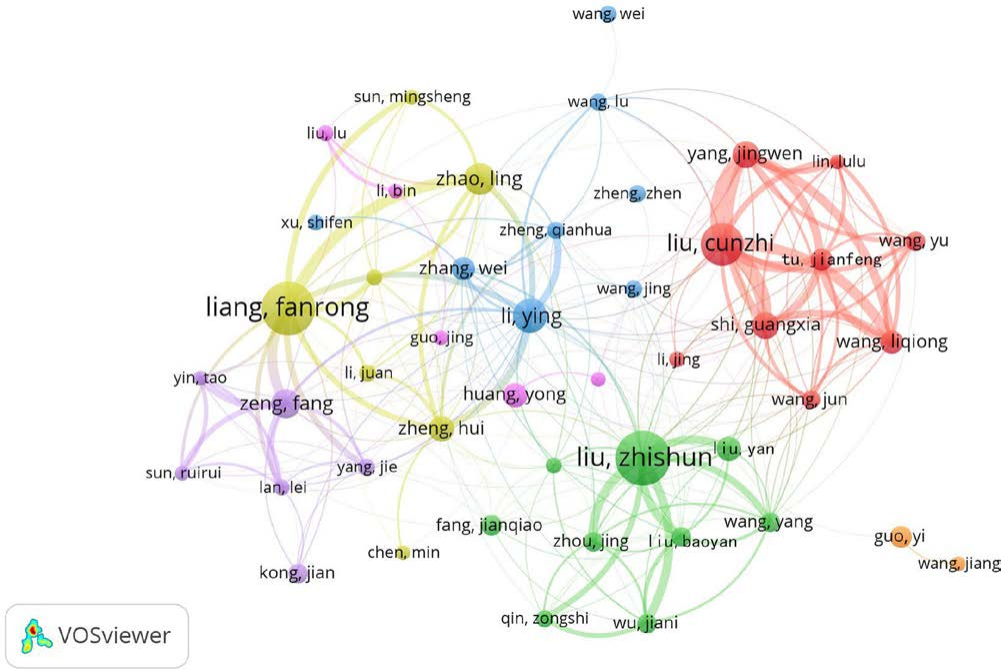
Network map of the co-occurrence of Chinese authors on clinical SCI associated with acupuncture and moxibustion from 2013 to 2022. SCI = Scientific Citation Index.

**Figure 5.**
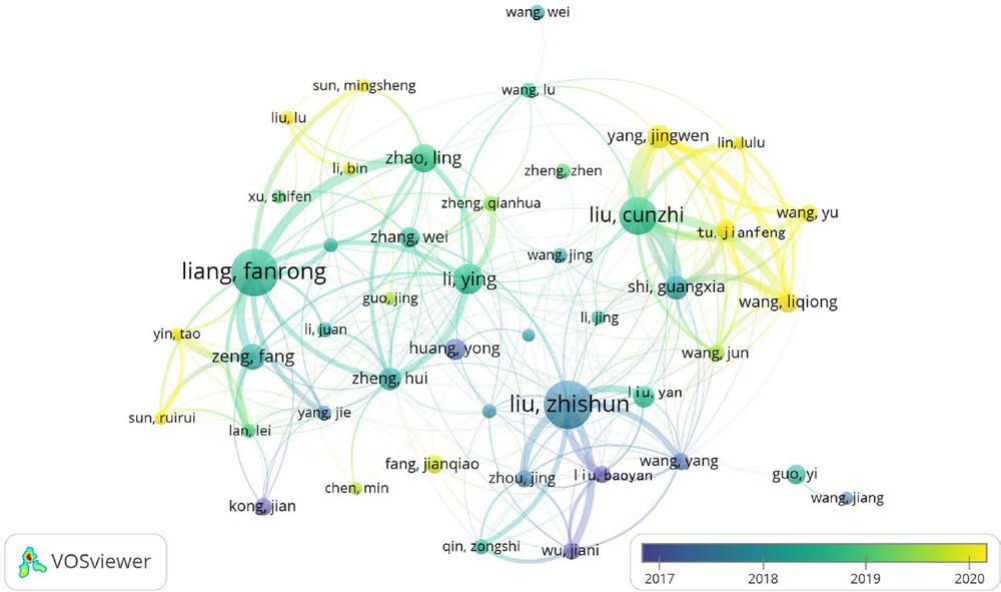
Network map of the co-occurrence of Chinese authors on clinical SCI associated with acupuncture and moxibustion from 2013 to 2022. SCI = Scientific Citation Index.

The VOSviewer parameter is configured to 1.6.19, with a minimum publication threshold of 18 for Chinese authors. Consequently, the cooperative network graph and the author cooperative network timeline graph for 46 Chinese authors have been generated, as illustrated in Figures 4 and 5. As shown in Figure 4, Chinese authors can be roughly divided into 7 cooperative sets: the green set, represented by Liu Zhishun and Liu Baoyan, and the yellow set, represented by Liang Fanrong and Zhao Ling. The red set corresponds to Liu Cunzhi and Yang Jingwen, the blue set corresponds to Li Ying and Zhang Wei, the purple set corresponds to Zeng Fang and Yang Jie, and the pink set corresponds to Huang Yong. The orange set is represented by Guo Yi. Among them, Li Ying has been associated with 26 authors and has worked closely with 5 collection teams.

Figure 5 illustrates that circles with warm and cold color changes represent the year distance. As depicted in the figure, Liu Zhishun’s team was mostly concentrated around 2017, while Liang Fanrong’s team and Liu Cunzhi’s team were mostly focused between 2018 and 2019. Recently, the most prominent and active teams have been the red ensembles of Yang Jingwen, Tu Jianfeng, and Wang Liqiong.

### 3.6. Main countries/regions for global clinical acupuncture SCI literature output

A total of 4554 SCI publications from 63 countries or regions were included. Among the included publications, 2503 originated from Mainland China, representing 45.97% of the total publications. In the past decade, the United States produced 872 SCI papers on acupuncture, while South Korea contributed 450. The top 3 countries in terms of publication volume over the last 10 years accounted for 70.25% of the total literature included. Additionally, Australia, the United Kingdom, Taiwan, and Germany all have publication volumes exceeding 100 papers, as detailed in Table 5.

**Table 5:** Top 10 countries and regions in terms of the number of clinical acupuncture SCI publications from 2013 to 2022

Country	The number of publications	The proportion of publications
PEOPLES R CHINA	2503	45.97%
USA	872	16.01%
SOUTH KOREA	450	8.26%
AUSTRALIA	199	3.65%
UK	192	3.53%
TAIWAN	178	3.27%
GERMANY	145	2.66%
BRAZIL	89	1.63%
JAPAN	86	1.58%
CANADA	80	1.47%
Total	4794	88.03%

SCI = Scientific Citation Index.

**Figure 6.**
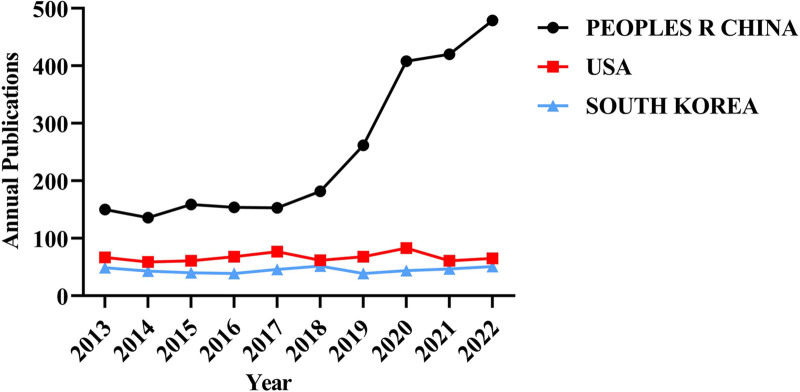
Annual publication trends of the top 3 countries in clinical acupuncture SCI publications. SCI = Scientific Citation Index.

As shown in Figure 6 a comprehensive analysis of the publication trends of acupuncture SCI papers published over the past decade reveals that since 2013, China’s annual publication volume has led that of the United States, South Korea, and other regions. Both South Korea and the United States have exhibited a stable trend in the number of acupuncture SCI publications over the past decade. The annual publication trend in China was relatively stable prior to 2018, followed by a rapid increase in publication volume from 2018 to 2020. Following 2020, the trend has shown moderation while still remaining on a comparatively high growth trajectory. Concerning the development of acupuncture SCI publications in mainland China itself, the number of publications in 2022 has reached 479, which is 3 times greater than the volume in 2013 and 7 times greater than that in the United States in the same year. This underscores the substantial efforts and contributions made by Mainland China in the field of acupuncture.

**Figure 7.**
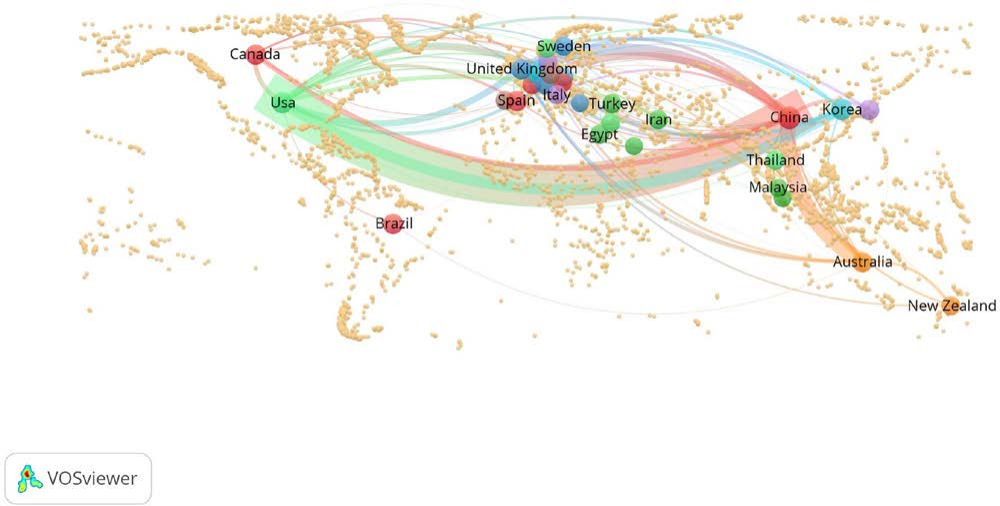
Geographical cooperation map of countries or regions in clinical acupuncture SCI publications from 2013 to 2022. SCI = Scientific Citation Index.

Using VOSviewer 1.6.19, the minimum number of publications required for countries or regions was set to 6. In the accompanying Excel spreadsheet, the latitude and longitude of each country are indicated; Taiwan and Mainland China are consolidated as China, while Scotland, England, Northern Ireland, and Wales are grouped as the United Kingdom. A global geographical cooperation map encompassing 31 countries or regions was generated, as illustrated in Figure 7. In the figure, circles represent countries, and lines represent cooperation between countries; the thicker the line is, the closer the cooperation between the 2 countries. China and the United States engage in the most frequent exchanges regarding acupuncture, with both countries placing particular emphasis on cooperation with other nations or regions. The link strength for China is 533, while the link strength for the United States is 440. Therefore, although the number of acupuncture SCI publications in the United States over the past decade has been much lower than that in China, the number of published SCI papers has greater international impact. Figure 7 shows that the countries or regions included in the literature are primarily concentrated in Europe, East Asia, and North America, with European countries representing the highest proportion.

### 3.7. The main source institutions for SCI literature in acupuncture clinical studies

A total of 3275 institutions were analyzed in the study, with the top 10 institutions by output detailed in Table 6. Among these, 8 institutions are based in China, whereas 2 are situated in Korea. This underscores the substantial contribution of Chinese and Korean research institutions to advancing acupuncture research. This study also emphasizes the considerable efforts undertaken by traditional Chinese medicine universities in China to modernize acupuncture practices.

**Table 6:** Top 10 institutions with the highest number of published SCI papers on acupuncture clinical research from 2013 to 2022

Institution name	Number of dispatches	Country of origin
Beijing University of Chinese Medicine	360	China
Chengdu University of Traditional Chinese Medicine	326	China
Guangzhou University of Chinese Medicine	274	China
China Academy of Chinese Medical Sciences	261	China
Kyung Hee University	237	South Korea
Shanghai University of Traditional Chinese Medicine	175	China
Capital Medical University	163	China
Tianjin University of Traditional Chinese Medicine	147	China
Korea Institute of Oriental Medicine Kiom	143	South Korea
Guang Anmen Hospital Cacms	122	China

SCI = Scientific Citation Index.

**Figure 8.**
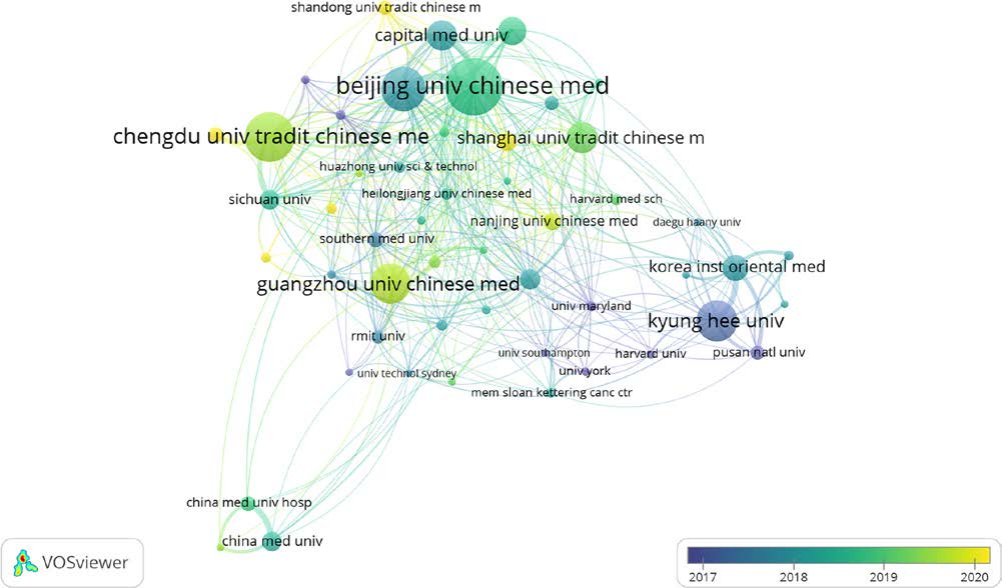
2013 to 2022 A time-elapsed collaboration map of SCI-Source institutions in acupuncture clinical research. SCI = Scientific Citation Index.

Setting the minimum number of publications per research institution in VOSviewer 1.6.19 to 28 yielded a total of 50 institution-time collaboration maps. Figure 8 depict extensive collaboration among institutions from various countries or regions worldwide. Korean institutions engaged in active collaboration from 2017 to 2018, while Chinese research institutions became notably active after 2018. Particularly active recently are Shandong University of Traditional Chinese Medicine, Changchun University of Chinese Medicine, Zhejiang University of Chinese Medicine, and Jiangxi University of Chinese Medicine, as highlighted in the figures.

### 3.8. Keyword analysis

#### 3.8.1. Keyword density analysis

**Figure 9.**
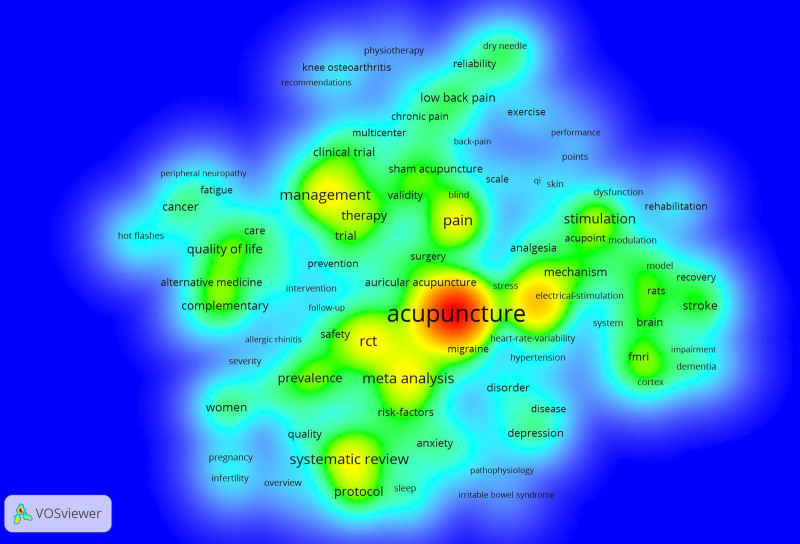
SCI keyword density map of acupuncture clinical research from 2013 to 2022. SCI = Scientific Citation Index.

In the literature, the author and plus keywords were combined, resulting in a total of 9028 keywords. Using a VOSviewer 1.6.19 parameter of 23 for minimum keyword appearances generated 200 density maps, as illustrated in Figure 9. In these maps, larger keyword labels appear more red, indicating higher weights, whereas smaller labels appear more blue, indicating lower weights. Therefore, keywords in the red and yellow regions of these density maps highlight research that has concentrated on acupuncture over the past decade. Acupuncture is prominently featured in the red area, indicating the highest weight; electroacupuncture is located in the deep yellow area, ranking second; pain, management, meta-analysis, randomized controlled trial (RCT), and systematic review are positioned in the yellow area, ranking third; while stimulation, functional magnetic resonance imaging (fMRI), mechanism, complementary, protocol, and prevalence are found in the light yellow area, ranking fourth.

The 30 keywords with the highest frequency of occurrence are listed in Table 7. Among these, acupuncture had the highest frequency of occurrence (2704), followed by electroacupuncture (899), which ranked second. Pain ranked third, with a frequency of 584, while management followed closely with a frequency of 580. In addition, meta-analyses, RCTs, systematic reviews, and protocols are the most common research methods used in acupuncture studies, whereas quality of life, mechanism, stroke, low back pain, cancer, depression, fMRI, sham acupuncture, and placebo are the most common disease, content, and mechanism research methods used in acupuncture studies.

**Table 7:** Top 30 high-frequency keywords in acupuncture clinical SCI studies from 2013 to 2022

Number	Keywords	Frequency of occurrence	Number	Keywords	Frequency of occurrence
1	acupuncture	2704	16	mechanism	208
2	electroacupuncture	899	17	stroke	205
3	pain	584	18	clinical trial	203
4	management	580	19	low back pain	186
5	Meta-analysis	541	20	trial	186
6	RCT	528	21	cancer	182
7	systematic review	510	22	risk-factors	171
8	stimulation	366	23	depression	170
9	protocol	321	24	safety	170
10	efficacy	289	25	validity	170
11	prevalence	284	26	alternative medicine	168
12	therapy	282	27	fMRI	164
13	quality of life	268	28	sham acupuncture	151
14	women	236	29	placebo	147
15	complementary	226	30	quality	142

SCI = Scientific Citation Index.

#### 3.8.2. Keyword clustering analysis

**Figure 10.**
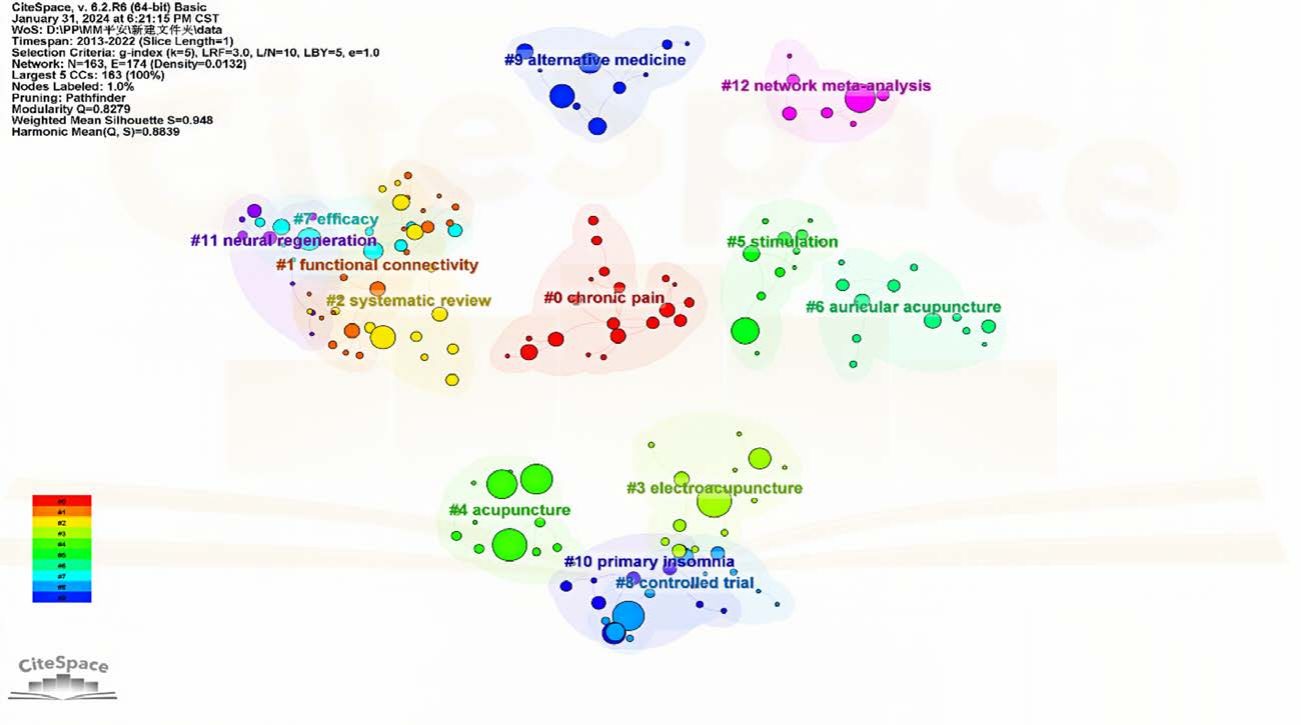
Clustering map of SCI keywords in acupuncture clinical research from 2013 to 2022. SCI = Scientific Citation Index.

CiteSpace 6.2 was utilized for the analysis. For R6, the number of years per slice was set to 1, the g index was set to 5, and the keywords were standardized to generate a keyword co-occurrence map, as illustrated in Figure 10. After the software analysis of the 4554 articles in this study, they were categorized into 13 keyword clusters. They were as follows: #0, chronic pain; #1, functional connectivity; #2, systematic review; #3, electroacupuncture; #4, acupuncture; #5, stimulation; #6, auricular acupuncture; #7, efficacy; #8, controlled trial; #9, alternative medicine; #10, primary insomnia; #11, neural regeneration; and #12, network meta-analysis. The clustered data were exported to the network_summary_table spreadsheet and supplemented with specific information using Cluster Explorer, as presented in Tables 8 and 9.

**Table 8:** Specific information on SCI keyword clustering for acupuncture clinical research from 2013 to 2022

Cluster ID	Silhouette	Relevant terms
0	0.961	multicenter; chronic pain; sham acupuncture; placebo; double-blind; clinical trial; validation; pain management; acupuncture analgesia; back pain; cancer
1	1	fMRI; brain; functional connectivity; cortex; brain networks; migraine; default mode network; dementia; electroacupuncture stimulation; Alzheimer’s disease; sympathetic nerve activity;
2	0.966	systematic review; outcome; knee osteoarthritis; neuropathic pain; surgery; in vitro fertilization; insomnia; functional dyspepsia; hip; therapy
3	0.815	expression; activation; ovulation; electroacupuncture; women; polycystic ovary syndrome; pregnancy; inflammation; frequency; cognitive impairment; human brain
4	1	acupuncture; dry needling; management; exercise; disability; neck pain; pain; mechanism; trigger point; dysfunction; association; performance
5	1	analgesia; irritable bowel syndrome; modulation; stimulation; relief; heart rate variability; blood pressure; acupuncture treatment; consensus; dissociation; epidemiology
6	0.929	quality; auricular acupuncture; fatigue; postoperative pain; risk factors; interventions; traditional Chinese medicine; stress; oxidative stress;
7	0.917	efficacy; prophylaxis; prevention; burden; safety; disease; blind; severity; headache; children
8	1	low back pain; randomized controlled trial; bibliometric analysis; osteoarthritis; guideline; index; study protocol; meta-analysis
9	1	breast cancer; vasomotor symptoms; hot flashes; complementary; care; quality of life; chemotherapy; postmenopausal; women
10	0.878	primary insomnia; health; validity; medicine; prevalence; reliability; scale; population; risk; Parkinson’s disease
11	0.921	recovery; connectivity; stroke; ischemic stroke; rehabilitation; nerve regeneration
12	0.978	anxiety; depression; disorders; network meta-analysis; responses

SCI = Scientific Citation Index.

**Table 9:** Specific information on high-centered keywords in acupuncture clinical SCI from 2013 to 2022

Number	Keywords	Centrality	Number	Keywords	Centrality
1	Acupuncture	0.86	19	Complementary	0.04
2	Electroacupuncture	0.13	20	Low back pain	0.04
3	Efficacy	0.09	21	Meta-analysis	0.04
4	fMRI	0.09	22	Quality	0.04
5	Management	0.08	23	Symptoms	0.04
6	Mechanism	0.08	24	Women	0.04
7	Brain	0.07	25	Functional connectivity	0.04
8	Depression	0.07	26	Activation	0.03
9	Pain	0.07	27	Alternative medicine	0.03
10	Therapy	0.07	28	Anxiety	0.03
11	Randomized controlled trial	0.07	29	Cortex	0.03
12	Sham acupuncture	0.06	30	Expression	0.03
13	Stimulation	0.06	31	Health	0.03
14	Disease	0.05	32	Medicine	0.03
15	Outcome	0.05	33	Prevalence	0.03
16	Validation	0.05	34	Recovery	0.03
17	Chronic pain	0.04	35	Cancer	0.03
18	Clinical trial	0.04			

SCI = Scientific Citation Index.

The keywords with a centrality greater than 0.2 are listed in Table 9. It is generally believed that keywords with a centrality greater than 0.1 have greater research value as nodes in some fields. The highest centrality for acupuncture was 0.86, while for electroacupuncture, it was 0.13, highlighting the significant role of electroacupuncture as a supplementary study within the international acupuncture field, demonstrating considerable clinical impact over the past decade. These high-centrality keywords focus on mechanistic studies, fMRI studies, and clinical efficacy studies from a research objective perspective. They also encompass RCTs and meta-analyses from a research methodology standpoint, addressing various disease types, such as lower back pain, acute and chronic pain, depression, anxiety disorders, female-related diseases, and cancer.

#### 3.8.3. Keyword timeline analysis

**Figure 11.**
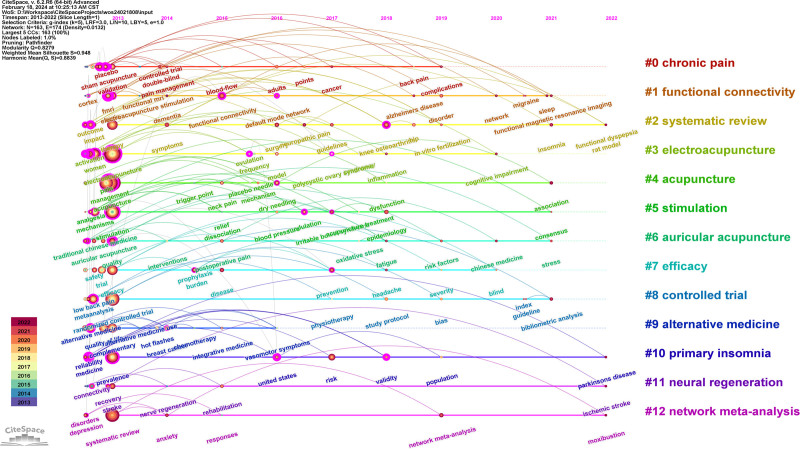
Time series chart of SCI keyword clustering in acupuncture clinical research from 2013 to 2022. SCI = Scientific Citation Index.

The keyword clustering timeline chart is generated based on the keyword clustering depicted in Figure 11, which illustrates the keywords associated with each clustering label over time, thereby providing a clearer representation of the evolution of research hotspots in acupuncture. As depicted in Figure 11, all 13 clusters had established research foundations before 2013, with robust beginnings for clusters #0 to #10 during the initial stages. In contrast, clusters #11 and #12 began with relatively weaker foundations. Before 2019, a wealth of dense research keywords was present, whereas core keywords after 2019 exhibited lower frequency and greater dispersion. Cluster #0 spans from 2013 to 2019, with the longest durations observed in Cluster #2, Cluster #10, Cluster #11, and Cluster #12, suggesting that these clusters possess ongoing potential and development trends for the future.

#### 3.8.4. Dynamic evolution of keywords

**Figure 12.**
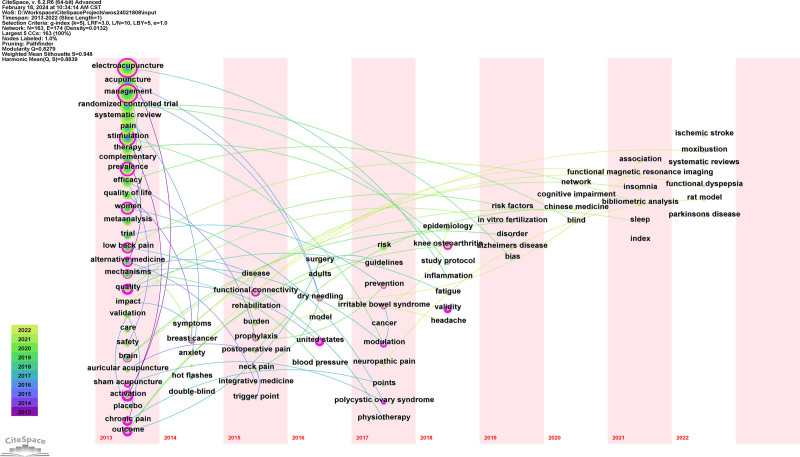
Evolution of SCI keywords in clinical acupuncture from 2013 to 2022 (time zone map). SCI = Scientific Citation Index.

Figure 12 show that the keywords related to pain appeared mostly before 2015, whereas the diseases studied after 2015 were related mostly to postoperative conditions. New keywords such as blood pressure, cancer, irritable bowel syndrome, fatigue, knee osteoarthritis, vasomotor symptoms, polycystic ovary syndrome, and pregnancy emerged after 2016 to 2018. Following the period from 2019 to 2022, additional keywords, including ischemic stroke, Alzheimer’s disease, dementia, Parkinson’s disease, cognitive impairment, effectiveness, and moxibustion, were introduced. The evolution of keywords over time suggests that acupuncture research extends beyond pain-related conditions, broadening disease applicability and increasing clinical use. Both clinical research and theoretical exploration have experienced innovation and development.

#### 3.8.5. Keyword sudden emergence detection

**Figure 13.**
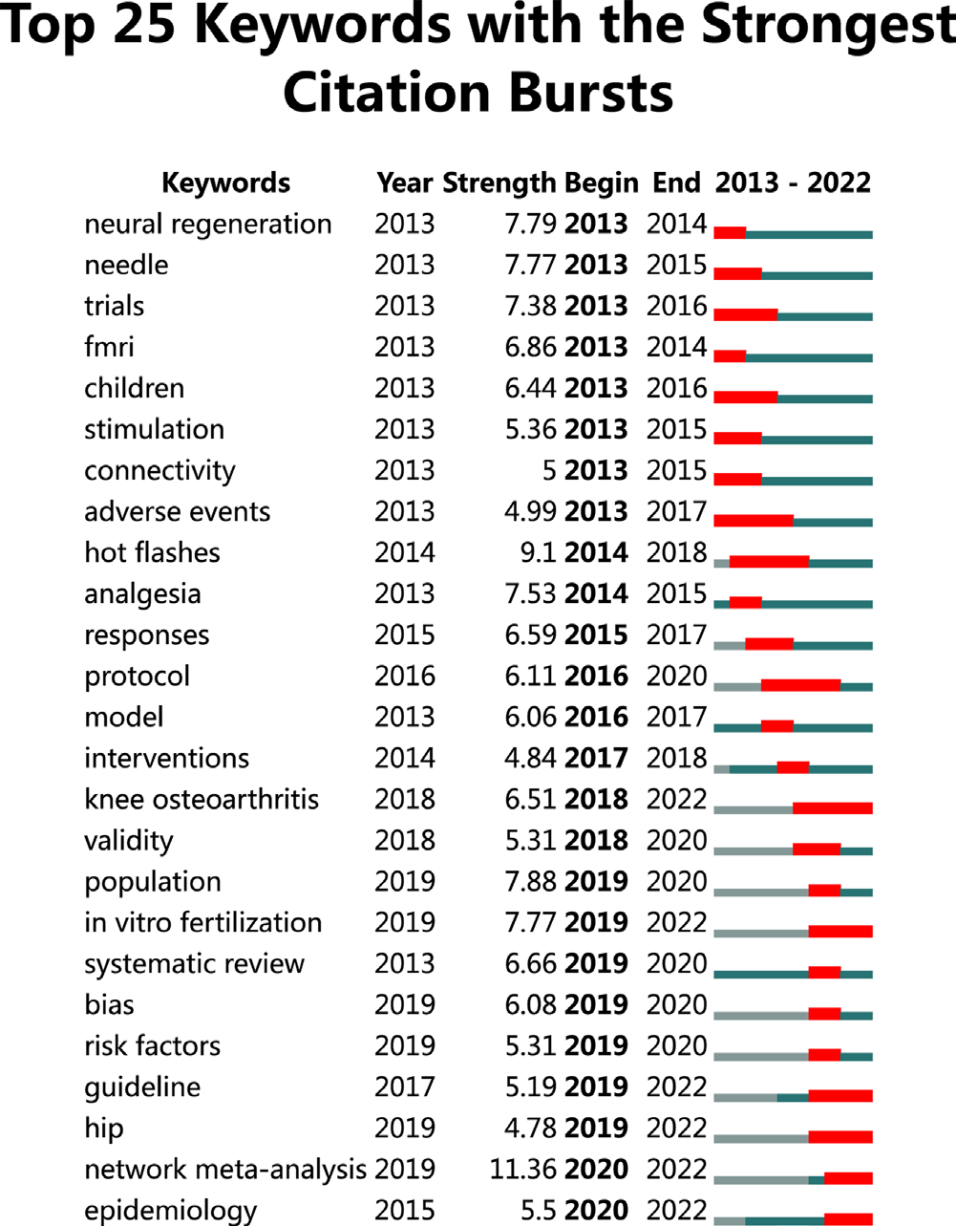
Topic emergence pattern of SCI literature on acupuncture clinical research from 2013 to 2022. SCI = Scientific Citation Index.

Figure 13 show a map of the top 25 most cited sudden keywords produced by CiteSpace, which reflects the terms that were frequently cited during a certain period of time. The red bars in the figure represent the emergence and duration of sudden keywords. The figure indicates that the keyword with the highest intensity is network meta-analysis, which emerged suddenly in 2020 and is expected to continue emerging until 2022. In addition, the keywords that emerged most frequently in 2022 are in the following order: in vitro fertilization, knee osteoarthritis, epidemiology, guidelines, and hip. The keywords with the longest duration of occurrence were hot flashes, which persisted from 2014 to 2018; knee osteoarthritis, lasting from 2018 to 2022; protocol, which extended from 2016 to 2020; and adverse events, which were present from 2013 to 2017.

### 3.9. Analysis of highly cited SCI papers in acupuncture

**Figure 14.**
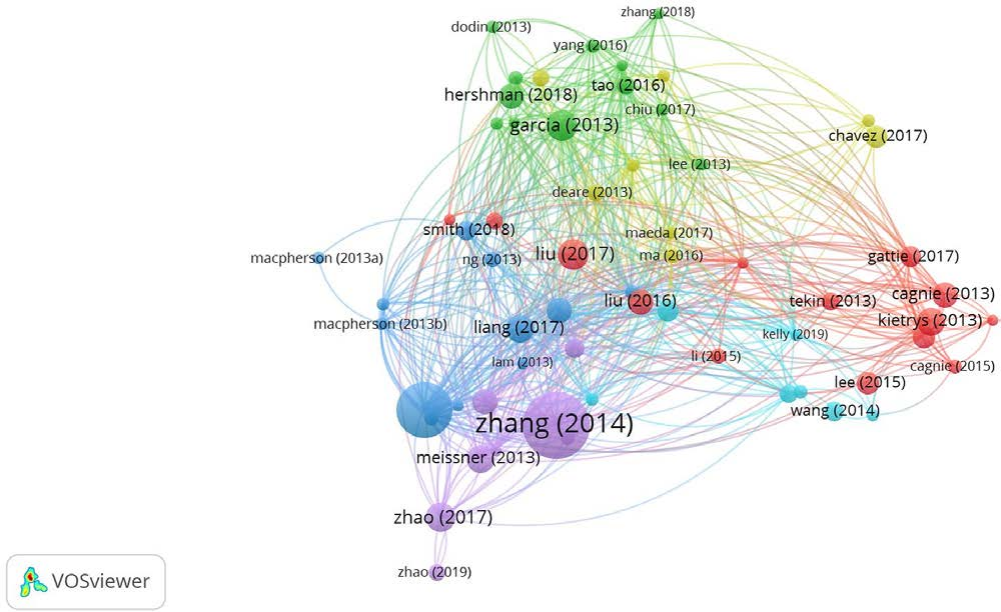
Cocitation map of highly cited clinical SCI literature on acupuncture from 2013 to 2022. SCI = Scientific Citation Index.

The fundamental details of the 10 most frequently cited SCI studies from 2013 to 2022 are presented in Table 10, where the total citation count is derived from the aggregation of all databases. The highest number of citations is 410, ranking 10th, whereas the 187th paper has the lowest number of citations. By setting the minimum citation count parameter in VOSviewer to 1.6.19–80, a cocitation network map of the 61 most cited studies was generated, as illustrated in Figure 14. The figure shows that the connections between highly cited studies are dense, the network is complex and stable, and the literature widely recognized by researchers also has close mutual citations, i.e., mutual recognition.

**Table 10:** Basic information of the highly cited SCI literature on acupuncture clinical research from 2013 to 2022

The title of the paper	Journal	Cited frequency	Year	The country
Acupuncture for Chronic Pain: Update of an Individual Patient Data Meta-Analysis	J Pain	410	2018	United States
Effect of Electroacupuncture on Urinary Leakage Among Women with Stress Urinary Incontinence A Randomized Clinical Trial	JAMA	295	2017	China
The Long-term Effect of Acupuncture for Migraine Prophylaxis A Randomized Clinical Trial	JAMA Intern Med	257	2017	China
Acupuncture for Chronic Knee Pain A Randomized Clinical Trial	JAMA	225	2014	Australia
Systematic Review of Acupuncture in Cancer Care: A Synthesis of the Evidence	J Clin Oncol	221	2013	United States
Acupuncture for Chronic Severe Functional Constipation A Randomized Trial	Ann Intern Med	216	2016	China
Study of acupuncture for low back pain in recent 20 years: a bibliometric analysis via CiteSpace	J Pain Res	208	2017	China
Effectiveness of Dry Needling for Upper-Quarter Myofascial Pain: A Systematic Review and Meta-analysis	J Orthop Sports Phys Ther	201	2013	United States
Effect of Acupuncture vs Sham Acupuncture or Waitlist Control on Joint Pain Related to Aromatase Inhibitors Among Women With Early-Stage Breast Cancer A Randomized Clinical Trial	JAMA	192	2018	United States
Acupuncture for the prevention of episodic migraine	Cochrane Database Syst Rev	187	2016	Germany

SCI = Scientific Citation Index.

Table 10 presents the top 10 papers on the use of acupuncture for treating pain-related diseases, including chronic pain, migraine, knee pain, low back pain, myofascial pain, and joint pain linked to aromatase inhibitors in early breast cancer patients. Additionally, 3 studies concentrated on the effects of acupuncture on stress urinary incontinence, cancer, and chronic severe functional constipation. The publication years consist of 2 studies from 2013, 1 from 2014, 2 from 2016, 3 from 2017, and 2 from 2018. The original countries included 4 papers from the United States, 4 from mainland China, 1 from Australia, and 1 from Germany. The majority of the papers were published in 1st-tier journals, except for J Pain Res (3rd-tier) and J Pain (2nd-tier). Notably, 3 studies were published in JAMA, contributed by researchers from China, the United States, and Australia, along with one study from JAMA Internal Medicine, originating from China. The presence of these studies in JAMA and JAMA Internal Medicine highlights the high academic significance and global recognition of research related to acupuncture.

### 3.10. Analysis of highly cited SCI literature in central acupuncture clinical research

The top 10 most highly cited articles with high centrality from 2013 to 2022 are listed in Table 11. Cited literature is the intellectual foundation of acupuncture research, and the progress of a field is developed on the basis of the cited literature. A centrality exceeding 0.1 signifies significant scientific contribution and research prominence.^[18]^ Among the 10 articles presented in the table, centrality values range from 0.37 (the highest) to 0.15 (the lowest), all exceeding the 0.1 threshold. The article with the highest centrality, titled “Inserting needles into the body: a meta-analysis of brain activity associated with acupuncture needle stimulation,” was published in the Journal of Pain in 2013 by a Korean acupuncturist and examined the meta-analysis of brain activity associated with acupuncture needle stimulation. Regarding the research content of the articles in Table 11, 6 articles discuss acupuncture for the treatment of pain, including 3 meta-analyses examining its effects on different types of chronic pain, a comparative study of true versus sham acupuncture in chronic pain treatment duration, an RCT on acupuncture for chronic knee pain, and an RCT on acupuncture for migraine prevention. Additionally, 2 articles focused on fMRI studies investigating the central mechanisms of acupuncture on brain function. The other articles comprised one study investigating the correlation between acupuncture sensation and efficacy, and another examining the effectiveness of acupuncture in the treatment of depression.

**Table 11:** Basic situation of clinical high-intensity SCI literature with a high number of citations related to acupuncture from 2013 to 2022

The title of the paper	Journal	Centrality	Year	Country or region
Inserting needles into the body: a meta-analysis of brain activity associated with acupuncture needle stimulation	J Pain	0.37	2013	South Korea
The benefit of combined acupuncture and antidepressant medication for depression: A systematic review and meta-analysis	J Affect Disord	0.36	2015	Taiwan, China
Acupuncture for chronic pain: individual patient data meta-analysis	Arch Intern Med	0.26	2012	United States
Acupuncture for chronic pain	JAMA	0.24	2014	United States
Acupuncture, the limbic system, and the anticorrelated networks of the brain	Auton Neurosci	0.21	2010	United States
Does the effect of acupuncture depend on needling sensation and manipulation?	Complement Ther Med	0.21	2013	South Korea
Acupuncture for chronic knee pain: a randomized clinical trial	JAMA	0.19	2014	Australia
The persistence of the effects of acupuncture after a course of treatment: a meta-analysis of patients with chronic pain	Pain	0.16	2017	United Kingdom
The Long-term Effect of Acupuncture for Migraine Prophylaxis: A Randomized Clinical Trial	JAMA Intern Med	0.15	2017	China
Acupuncture needling sensation: the neural correlates of deqi using fMRI	Brain Res	0.15	2010	United Kingdom

SCI = Scientific Citation Index.

According to our national-level analysis, these 10 articles were published in 5 countries, with 2 from China, 2 from the UK, 3 from the US, 2 from South Korea, and 1 from Australia. An analysis of publication dates indicated that 2 articles were published in 2010, 1 in 2012, 2 in 2013, 2 in 2014, 1 in 2015, and 2 in 2017. Within the journals where the articles appeared, 5 were categorized as top journals (JCR 1), including “Acupuncture for chronic pain” and “Acupuncture for chronic knee pain: a randomized clinical trial,” which were featured in the leading journal JAMA. The remaining 5 articles were spread across journals in categories 2 and 4, with 2 articles in each of those categories and 1 article in category 3.

### 3.11. Subject analysis of SCI literature studies on acupuncture clinical research

According to the literature, a comprehensive summary of 78 disciplines associated with acupuncture research has been statistically compiled over the past decade. The top 10 disciplines, ranked by the number of published articles, are presented in Table 12 in descending order. The complementary and alternative medicine field related to acupuncture research had the greatest number of articles published, totaling 1674 from 2013 to 2022. This discipline represents the primary focus of acupuncture research. Additionally, internal medicine related to acupuncture research is ranked second, with 881 articles published, while neuroscience and neurology related to acupuncture research is ranked third, with 617 articles published. Additionally, acupuncture researchers have been actively involved in oncology, health preservation and rehabilitation, gynecology, and anesthesiology. These fields have been focal points of acupuncture research over the past decade, indicating a trend toward multidisciplinary integration. A comparative analysis of research disciplines conducted annually indicated a steady rise in the number of articles associated with psychiatry since 2020 and a gradual increase in articles related to pharmacology since 2021, likely attributed to the “acupuncture and drug combination” theory. Furthermore, a significant increase in the number of articles related to mathematical computational biology was observed in 2022.

**Table 12:** Distribution of SCI research disciplines in acupuncture clinical studies from 2013 to 2022

Subject	Number
Integrative Complementary Medicine	1674
General Internal Medicine	881
Neurosciences Neurology	617
Research Experimental Medicine	346
Oncology	138
Science Technology Other Topics	130
Health Care Sciences Services	104
Rehabilitation	102
Anesthesiology	86
Obstetrics Gynecology	78

SCI = Scientific Citation Index.

## 4. Discussion

Statistical analysis revealed that the volume of SCI literature concerning acupuncture clinical studies has steadily increased over the past decade, spanning from 2013 to 2022. Over 45% of this literature was published in journals with impact factors ranging from 1 to 3. Notably, the journal with the greatest number of articles, Evid-Based Complement Alternat Med, is no longer indexed in the SCI database. The majority of SCI literature pertaining to acupuncture clinical studies continues to be published in journals that concentrate on “complementary and alternative medicine.” Among the top 10 journals by article count, only Acupuncture in Medicine is dedicated solely to acupuncture. This highlights acupuncture’s categorization as a therapy within “complementary and alternative medicine” globally, rather than mainstream medicine, with its clinical research currently characterized by modest quality and limited international influence.

China, the United States, and South Korea are the 3 leading countries contributing to the acupuncture SCI literature. The primary academic contributions are predominantly concentrated in China, the United States, South Korea, and the United Kingdom, with China taking the lead. The “Belt and Road Initiative” is anticipated to improve the integration of developing countries into international research collaborations.

Over 45% of the literature is published in journals with impact factors between 1 and 3. Most publications are in journals focused on “complementary and alternative medicine,” indicating that acupuncture is still considered a complementary therapy rather than mainstream medicine. However, significant achievements in acupuncture research have been made, especially in high-quality clinical studies and fMRI research. The recently published Blue Book on Acupuncture Clinical Research Evidence (2015–2024)^[1]^ by the World Federation of Acupuncture-Moxibustion Societies demonstrates that acupuncture not only plays a significant role in pain management,^[19]^ but also shows favorable effects in cancer patient rehabilitation,^[20]^ alleviation of adverse reactions to radiotherapy and chemotherapy, and improvement of quality of life. Additionally, acupuncture can regulate emotions and relieve anxiety and depression.^[21]^ Acupuncture is safe to use and easy to perform. Compared with pharmacological treatments, acupuncture does not cause drug dependence or adverse effects on vital organs such as the liver and kidneys. These studies not only provide substantial clinical evidence supporting the efficacy of acupuncture but also indicate its gradual integration into mainstream medical practice. The findings underscore acupuncture’s broad clinical applicability and serve as valuable references for future research directions.

China’s leading position in the output of clinical acupuncture SCI. Since 2013, China has been leading in the annual output of clinical acupuncture SCI, especially since 2018. Significant contributions to the field have been made by Chinese scholars, accompanied by an increasing number of high-quality publications.

However, an analysis of highly cited and highly central papers on acupuncture published in the past 10 years revealed that the achievements of acupuncture are still significant. In 2013, American researcher M Kay Garcia et al reported that acupuncture was a suitable adjunctive therapy for nausea and vomiting caused by cancer chemotherapy.^[22]^ In 2014, Rana S. Hinman et al reported that acupuncture therapy did not enhance pain or functional outcomes compared to sham acupuncture in the treatment of chronic knee pain.^[23]^ This finding sparked intense global debate and prompted international acupuncturists to investigate acupuncture treatment for knee pain. The most highly cited paper is a chronic pain study conducted by the Andrew J. Vickers team in 2018, which incorporated 10 additional high-quality trials with patient-level data related to chronic pain into the 2012 study. This research reached a new conclusion, indicating that the therapeutic effects of acupuncture would persist over time and could not be solely attributed to the placebo effect.^[21,24]^ Furthermore, the achievements of acupuncture are evident not only in international multicenter RCTs but also in several high-centrality fMRI studies that have examined the brain function responses elicited by acupuncture needle insertion into the human body.^[25–27]^ High-quality research outcomes can significantly influence the direction of acupuncture research, with high-quality clinical studies serving as the foundation for informing scientific decision-making and clinical guidelines. Despite the limitations present in certain trial designs, these studies have stimulated verification and reflection among many acupuncturists and clinicians. The contributions of these studies have far surpassed those of the trials themselves. Consequently, the advancement of acupuncture in the clinical domain has been impeded by the ongoing scarcity of high-quality evidence-based medicine. Moreover, verifying the clinical efficacy of acupuncture solely through RCTs is inadequate. Research methods that preserve the traditional theoretical paradigm of acupuncture while guided by the modern theoretical paradigm of acupuncture are urgently needed.^[28,29]^ Therefore, in the future, researchers will not only pursue the number of SCIs but also dedicate themselves to high-quality innovative research.

Based on an analysis of keyword density maps, high-frequency and high-centrality keywords, keyword clustering, highly cited literature, and co-cited literature, the primary emphasis remains on the analgesic effects of acupuncture and electrical acupuncture. Pain-related conditions predominantly include chronic issues such as lumbar pain, migraine, knee joint pain, and cervical pain. Knee osteoarthritis has consistently been a significant focus through 2022, demonstrating potential for further development. Since 2015, myofascial pain has gained increasing attention, with the use of dry needle stimulation of “trigger points” emerging as a notable new approach in pain management. Postoperative pain and pain induced by cancer chemotherapy have emerged as important areas of study over the past decade. Ear acupuncture is frequently employed to reduce preoperative stress, facilitate postoperative recovery, and alleviate accompanying symptoms such as fatigue. Electrical acupuncture has gained substantial clinical influence because of its ability to measure stimulation frequency and intensity, improve reproducibility, and its suitability for both basic and clinical research.^[30]^ It serves as a valuable complement to traditional acupuncture research.

Nonpain-related hot areas focus on female-related diseases, especially breast cancer, female stress urinary incontinence, polycystic ovary syndrome, and menopausal syndrome. In assisted reproduction, in vitro fertilization has remained a prominent keyword through 2022, indicating ongoing potential for future development. Diseases of the digestive system are primarily associated with functional constipation, functional dyspepsia, and irritable bowel syndrome. The neurological diseases of interest included ischemic stroke, anxiety and depression, insomnia, Alzheimer’s disease, Parkinson’s disease, and dementia.

Through research method analysis, key terms in acupuncture clinical research include epidemiology, RCT, bibliometric analysis (literature metric analysis), study protocol, guideline, meta-analysis, and network meta-analysis. RCTs have consistently been a central focus in acupuncture research methods, with over 14,000 acupuncture-related RCTs published globally. However, challenges continue to exist regarding their design and reporting quality, including the establishment of effective sham acupuncture controls, management of placebo effects, implementation of blind designs, and quantification of expectancy effects.^[31,32]^ The limitations in RCT evidence and quality have hindered the development of clinical practice guidelines for acupuncture, resulting in modest international practical value and recommendation levels.^[33]^ To ensure reliable conclusions and reproducible experimental procedures, researchers must adhere strictly to guidelines such as CONSORT and STRICTA.^[34]^ Continuous refinement of acupuncture protocols and translation of high-quality clinical evidence into robust practice guidelines are essential for enhancing the formulation and dissemination of acupuncture application guidelines in the future.^[32,35]^ Among the emerging keywords, network meta-analysis, a popular research method, stands out as the most intense and persistent keyword. This method extends traditional meta-analysis techniques, allowing for the quantification of effects from different interventions for treating the same disease, thereby facilitating the selection of the optimal treatment strategy. Clinical epidemiology, serving as a fundamental method of clinical research, has been widely applied in the field of acupuncture, generating a large amount of high-quality clinical evidence in conjunction with evidence-based medicine. However, with the trend of multidisciplinary convergence in the new era, artificial intelligence and mathematical computational biology have gradually appeared in clinical research. Promoting the scientific application of clinical epidemiology in acupuncture and innovating the paradigm of acupuncture clinical research is a problem that should be prioritized and addressed in the present and future.^[36]^

In addition to evaluating the effectiveness of acupuncture, safety has emerged as a prominent topic in the international acupuncture community, with “adverse events” becoming a notable keyword in recent years. Although acupuncture is widely regarded as a safe and environmentally friendly treatment method globally, improper administration can result in serious adverse reactions, including pneumothorax, spinal injury, and infection.^[37]^ Therefore, assessing the risks associated with adverse events from acupuncture is essential for clinical decision-making.^[38]^ A prospective observational study indicated that ensuring the safety of acupuncture treatment necessitates rigorous and standardized training for acupuncturists. Additionally, enhancing communication between patients and medical staff to obtain informed consent is essential for mitigating medical disputes.^[38]^

The concept of acupuncture for “preventing diseases before they occur,” also known as disease prevention, has gained significant attention. The World Health Organization emphasized in “Meeting the Challenge of the 21st Century” that health is a fundamental human right, shifting the focus of medical research toward overall human health and individual capacity for health discovery and development. Acupuncture, rooted in traditional theories of “regulating yin and yang” through benign two-way adjustment, aims to achieve balance by stimulating specific acupoints. This stimulation activates the body’s intrinsic protective mechanisms, potentially preventing the onset, progression, and deterioration of diseases. Current research on disease prevention through acupuncture primarily focuses on chronic noncommunicable diseases,^[39]^ yet understanding its preventive mechanisms remains in the early stages. As modern society increasingly values health and wellness, acupuncture, as a nonaddictive and nonpharmacological therapy, is poised to play a pivotal role in disease prevention.^[40]^ Therefore, future developments in this field should focus on clarifying the clinical efficacy of disease prevention, elucidating its scientific mechanisms, and establishing diagnostic and treatment standards that distinguish between diseases and non-diseases.^[41]^

## 5. Looking ahead and limitations

This study exclusively examines clinically relevant studies on acupuncture and does not encompass animal experimental research, thus limiting its exploration of acupuncture mechanisms. Additionally, excluding therapeutic methods such as laser acupuncture, transcutaneous electrical stimulation, magnetic stimulation, acupoint massage, acupoint paste application, massage combined with acupuncture, and cupping – each of which acts on acupoints or related areas – can be considered within the broader scope of “acupuncture.” Moving forward, it is essential to recognize these limitations and conduct comprehensive analysis and exploration within the field of acupuncture.

## 6. Conclusion

Over the past decade, the volume of SCI literature focused on acupuncture clinical studies has steadily increased, indicating a trend toward increasing international recognition, with China emerging as a leading contributor. The primary focus of research in acupuncture clinical studies continues to be acupuncture and electrical acupuncture for pain management. Future research directions with potential include acupuncture treatments for knee osteoarthritis, diseases related to cognitive disorders, and acupuncture-assisted reproduction.

## Acknowledgments

We appreciate the contributions of all participants, institutions or funds that assisted in the work.

## Author contributions

**Conceptualization:** Xiangdong Wang, Bo Li, Yuping Ma.

**Data curation:** Xiangdong Wang, Bo Li, Yuping Ma, Ying Cui, Xinming Yang, Yuxian Li, Anna Jing, Yutong Zhou, Mingyue Li, Sixuan Wang, Yufeng Tu.

**Software:** Xiangdong Wang, Xinming Yang, Yuxian Li, Anna Jing, Yutong Zhou.

**Visualization:** Xiangdong Wang, Yuping Ma, Ying Cui, Xinming Yang, Yuxian Li, Anna Jing, Yutong Zhou, Mingyue Li, Sixuan Wang, Yufeng Tu.

**Writing – original draft:** Xiangdong Wang, Bo Li, Yuping Ma, Ying Cui.

**Writing – review & editing:** Xiangdong Wang, Bo Li, Yuping Ma, Ying Cui.
